# Mutagenesis of *Trichoderma reesei* endoglucanase I: impact of expression host on activity and stability at elevated temperatures

**DOI:** 10.1186/s12896-015-0118-z

**Published:** 2015-02-21

**Authors:** Harshal A Chokhawala, Christine M Roche, Tae-Wan Kim, Meera E Atreya, Neeraja Vegesna, Craig M Dana, Harvey W Blanch, Douglas S Clark

**Affiliations:** Energy Biosciences Institute, University of California, Berkeley, CA 94720 USA; Department of Chemical and Biomolecular Engineering, University of California, Berkeley, CA 94720 USA; Department of Biotechnology, Indian Institute of Technology, Kharagpur, West Bengal 721301 India; Department of Chemistry, University of California, Berkeley, CA 94720 USA

**Keywords:** *Trichoderma reesei*, Endoglucanase I, B-factor, Cellulase, Thermostability, Cell-free synthesis, *Saccharomyces cerevisiae*, *Neurospora crassa*, Pyroglutamate, Glutaminyl cylcase

## Abstract

**Background:**

*Trichoderma reesei* is a key cellulase source for economically saccharifying cellulosic biomass for the production of biofuels. Lignocellulose hydrolysis at temperatures above the optimum temperature of *T. reesei* cellulases (~50°C) could provide many significant advantages, including reduced viscosity at high-solids loadings, lower risk of microbial contamination during saccharification, greater compatibility with high-temperature biomass pretreatment, and faster rates of hydrolysis. These potential advantages motivate efforts to engineer *T. reesei* cellulases that can hydrolyze lignocellulose at temperatures ranging from 60–70°C.

**Results:**

A B-factor guided approach for improving thermostability was used to engineer variants of endoglucanase I (Cel7B) from *T. reesei* (TrEGI) that are able to hydrolyze cellulosic substrates more rapidly than the recombinant wild-type TrEGI at temperatures ranging from 50–70°C. When expressed in *T. reesei*, TrEGI variant G230A/D113S/D115T (G230A/D113S/D115T Tr_TrEGI) had a higher apparent melting temperature (3°C increase in T_m_) and improved half-life at 60°C (t_1/2_ = 161 hr) than the recombinant (*T. reesei* host) wild-type TrEGI (t_1/2_ = 74 hr at 60°C, Tr_TrEGI). Furthermore, G230A/D113S/D115T Tr_TrEGI showed 2-fold improved activity compared to Tr_TrEGI at 65°C on solid cellulosic substrates, and was as efficient in hydrolyzing cellulose at 60°C as Tr_TrEGI was at 50°C. The activities and stabilities of the recombinant TrEGI enzymes followed similar trends but differed significantly in magnitude depending on the expression host (*Escherichia coli* cell-free, *Saccharomyces cerevisiae*, *Neurospora crassa,* or *T. reesei*). Compared to *N.crassa*-expressed TrEGI, *S. cerevisiae*-expressed TrEGI showed inferior activity and stability, which was attributed to the lack of cyclization of the N-terminal glutamine in Sc_TrEGI and not to differences in glycosylation. N-terminal pyroglutamate formation in TrEGI expressed in *S. cerevisiae* was found to be essential in elevating its activity and stability to levels similar to the *T. reesei* or *N. crassa*-expressed enzyme, highlighting the importance of this ubiquitous modification in GH7 enzymes.

**Conclusion:**

Structure-guided evolution of *T. reesei* EGI was used to engineer enzymes with increased thermal stability and activity on solid cellulosic substrates. Production of TrEGI enzymes in four hosts highlighted the impact of the expression host and the role of N-terminal pyroglutamate formation on the activity and stability of TrEGI enzymes.

**Electronic supplementary material:**

The online version of this article (doi:10.1186/s12896-015-0118-z) contains supplementary material, which is available to authorized users.

## Background

Efficient saccharification of cellulosic biomass to fermentable sugars is necessary for economical production of second generation biofuels. Several enzymes (exoglucanases, endoglucanases, oxidative enzymes, and β-glucosidases) act in concert to hydrolyze cellulose to glucose. The industrially employed filamentous fungus *Trichoderma reesei* has been evolved for cellulase production and is capable of secreting large quantities of cellulolytic enzymes at a relatively low cost [1,2]. These include two exoglucanases, Cel7A and Cel6A (CBH I and CBH II), which represent 50% and 20% of the total cellulase content, respectively, four endoglucanases, Cel7B (EGI), Cel5A (EGII), Cel12A (EGIII), and Cel45A (EGV) that represent approximately 15%, 10%, 1%, and <1% of the total cellulase content, respectively, and several lytic polysaccharide monooxygenases [3,4].

Lignocellulose hydrolysis at relatively high temperatures (above 50°C, which is near the optimum for many fungal cellulases) may offer potential advantages, including reduced solution viscosity at high-solids loadings (>20 wt%), lower risk of microbial contamination during saccharification, greater compatibility with high-temperature pretreatments, and potentially faster rates of hydrolysis [5]. The short half-lives of *T. reesei* cellulases at temperatures above 50°C, together with very low expression levels of thermophilic cellulases (typically less than 100 mg/L, compared to over 150 gm/L for *T. reesei* cellulases) [6], motivates the development of thermostable *T. reesei* cellulases that can hydrolyze lignocellulose efficiently at temperatures beyond 50°C. *T. reesei* endoglucanase I (TrEGI) is known to randomly cleave internal cellulosic bonds, thereby creating shorter cellulosic chains. Recent studies on optimizing the components of cellulase systems have underscored the importance of having TrEGI comprise a high fraction of the cellulase mixture (25–35%) in order to efficiently hydrolyze pretreated lignocellulosic biomass [7,8].

**Figure 1:**
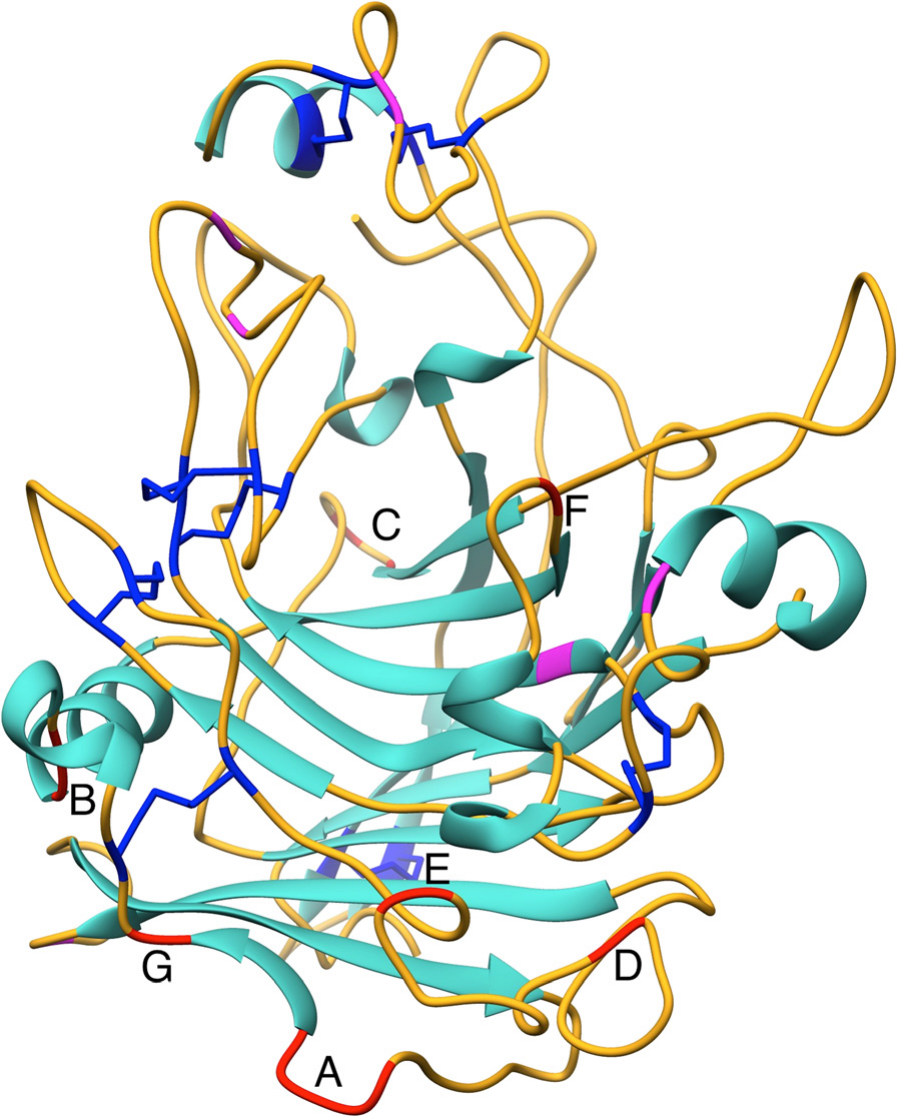
**Crystal structure of Endoglucanase I from** ***Trichoderma reesei*** **.** Disulfide bonds are shown in blue. N-glycosylation sites are shown in magenta. Mutagenesis sites are shown in red and are labeled as follows: A (aa 284–287), B (aa 301–302), C (aa 113, 115), D (aa 238), E (aa 230), F (aa 323), and G (aa 291). Mutations at site C and site E resulted in improved TrEGI enzyme variants. PDB code 1EG1.

TrEGI has a bimodular structure with a 375-amino-acid (aa) catalytic domain (CD, Figure 1) attached to a 35-aa carbohydrate-binding module (CBM) via a 26-aa linker. TrEGI has 11 disulfide bonds (8 in the CD [highlighted in blue in Figure 1] and 3 in the CBM), 6 N-glycosylation sites in the CD (highlighted in magenta in Figure 1), and a linker region rich in serine and threonine that are potential O-glycosylation sites. It was selected for engineering enhanced thermostability because of its importance in cellulose hydrolysis and the availability of its crystal structure [9].

**Figure 2:**
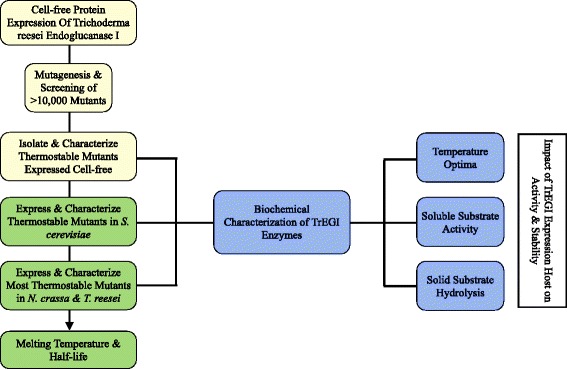
**Engineering thermostable Endoglucanase I from** ***Trichoderma reesei*** **.**

Although protein engineering has been used to improve the thermostability of numerous cellulases [10-13], there are no reports on improving the thermostability or increasing the optimal temperature of TrEGI. In this study, we employed a B-factor guided approach [14] to engineer thermostable variants of TrEGI using *E. coli* cell-free protein synthesis due its ease and throughput (Figure 2). Many thermostable mutants of TrEGI identified using cell-free protein synthesis were subsequently expressed in the fungal hosts *S. cerevisiae, N. crassa,* and *T. reesei* to better mimic the properties of the native TrEGI enzyme in terms of folding and glycosylation (Figure 2). TrEGI variants, particularly G230A/D113S/D115T, were more stable compared to their recombinant wild-type versions when both the variant and wild-type were expressed in *S. cerevisiae, N. crassa*, or *T. reesei,* indicating that the positive influence of these mutations on stability translates across different expression hosts*.* However, the *S. cerevisiae*-expressed enzymes were inferior in terms of activity and stability compared to the same enzymes expressed in *T. reesei* or *N. crassa*, which we attribute to the lack of cyclization of the N-terminal glutamine of TrEG1 expressed in our *S. cerevisiae* system.

## Results and discussion

### B-factor guided mutagenesis

Our efforts to improve the thermostability of TrEGI entailed a B-factor guided approach (B-FIT method) [14]. The B-factor is a measure of the average deviation of a given residue from its mean position in the crystal structure. Residues having the highest average B-factors (obtained from the crystal structure) correspond to the most flexible sites in the protein and provide targets for mutagenesis to improve thermostability. This strategy is based on the premise that the thermostability of a mesophilic enzyme can be enhanced by increasing its rigidity at the most flexible sites in its structure. Examples of B-factor guided mutagenesis to increase thermostability include a *Bacillus subtilis* lipase ($\varDelta {T}_{50}^{60}$ of 42°C) [14] and a *Pseudomonas fluorescens* esterase ($\varDelta {T}_{50}^{60}$ of 9°C) [15], where $\varDelta {T}_{50}^{60}$ corresponds to the change in temperature at which the enzyme loses half its activity after an hour of incubation.

Using the crystal structure of the TrEGI catalytic domain (Figure 1), we identified the 20 amino acids with the highest B-factors. These amino acids were grouped in seven sites (Figure 1, in red) comprising one or more amino acids [Site A (aa 284–287), Site B (aa 301–302), Site C (aa 113, 115), Site D (aa 238), Site E (aa 230), Site F (aa 323), Site G (aa 291)], and were chosen for site-saturation mutagenesis. Amino acids with high B-factors that were in close proximity to the N- or C-terminus, disulfide bridges, or N-glycosylation sites in the TrEGI crystal structure were not selected because they could interfere with disulfide-bond formation or lead to false positives due to compensation for non-glycosylation in a bacterial expression system.

A modification of the cell-free protein synthesis protocol developed in our laboratory [16] was used to express TrEGI in an active, soluble form (Additional file 1: Figure S1). The stability of the cell-free expressed TrEGI is comparable to TrEGI expressed in *E. coli* [17], but is considerably reduced compared to the native enzyme expressed in *T. reesei* [18]. Although cell-free protein synthesis yielded an inferior TrEGI enzyme, we proceeded to use cell-free expression as a tool to rapidly screen mutants of TrEGI, with the assumption that the effect of mutations imparting thermostability will be conserved upon expression in fungal hosts such as *T. reesei*.

The protocol for screening and selection of mutant TrEGI enzymes is shown in (Additional file 1: Figure S2). Mutagenesis and screening of 11,000 mutants at sites A-G generated ~500 mutants with higher activity toward carboxymethyl cellulose (CMC) after pre-incubation at 50°C for 45 min than the wild-type TrEGI expressed using cell-free synthesis. Sequencing the 70 mutants that showed highest activities after the heat treatment revealed that all but six mutants had a mutation at site E (amino acid 230, glycine). Alanine, arginine, serine, threonine, leucine, lysine, glutamic acid, glutamine, and methionine substitutions at this position all imparted improved thermostability. In addition, we found mutations at site C (amino acids D113, D115) that gave rise to more stable variants of TrEGI. Amino acid changes at this site for these TrEGI mutants were D113S/D113T and D115L/D115G. Even though the D113L/D115G mutant had greater thermostability at 50°C, it was not pursued further because it had low specific activity relative to wild-type TrEGI expressed using cell-free synthesis. Since the single mutants (D113S, and D115T) were not isolated during the screening process that covered >95% of all possible amino acid combinations at site C [19], it is highly likely that mutations at both these sites need to be present simultaneously to afford increased stability.

### Activity measurements of round I variants with improved thermostability

**Figure 3:**
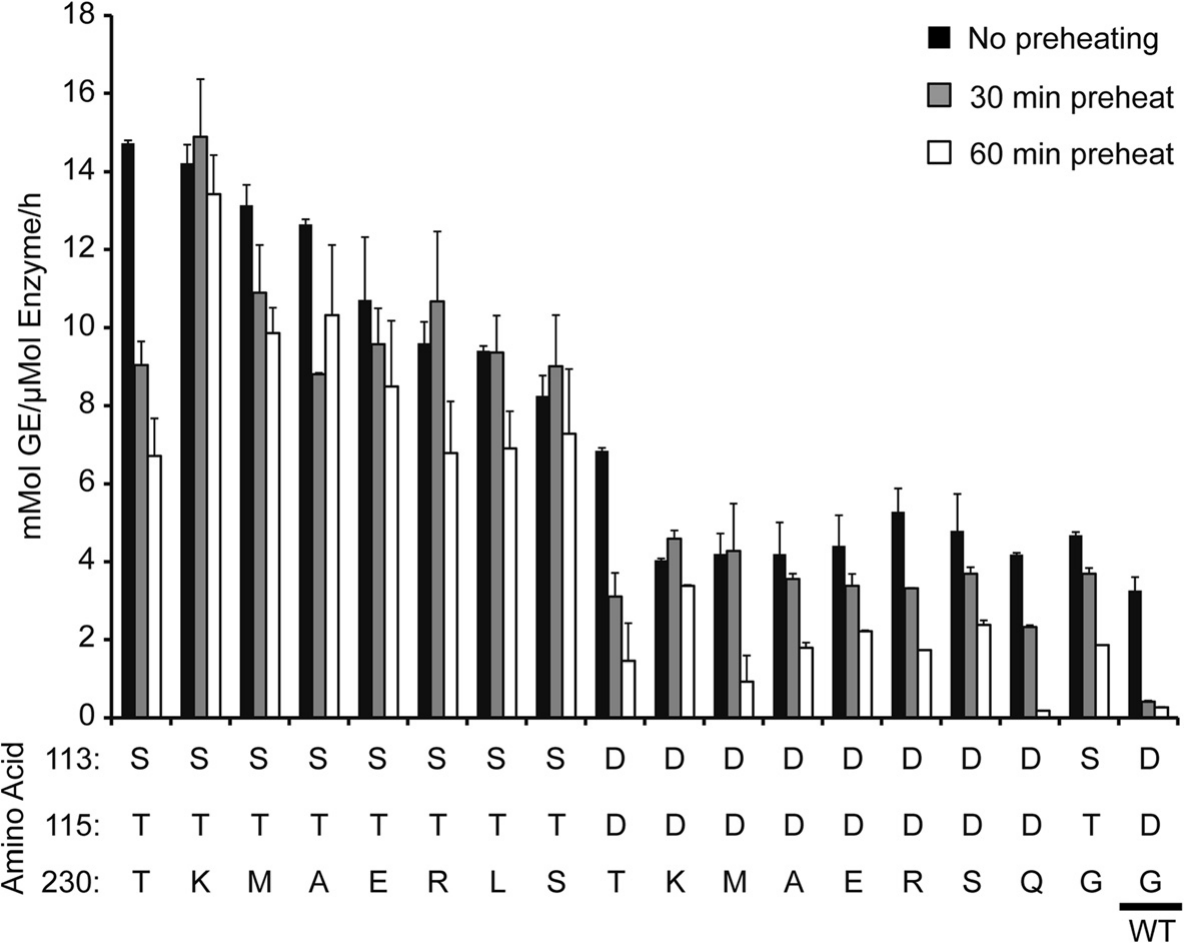
**Activity of** ***T. reesei*** **endoglucanase I mutants at 50°C on CMC.** Error bars represent one standard deviation. TrEGI proteins were expressed using cell-free protein synthesis and were used without any purification.

The activities of some of these thermostable TrEGI mutants toward CMC, based on the amount of reducing sugar released before and after exposure to 50°C for various times, are summarized in Figure 3. Combining mutations at G230 with mutations at sites D113 and D115 resulted in triple mutant enzymes (G230X/D113S/D115T, X = K, A, S, R, E, L, T, M; Figure 3) that were more active at 50°C than either of the engineered parents (Figure 3). All the triple mutants showed higher specific activity at 50°C on CMC compared to the wild-type TrEGI expressed using cell-free synthesis at 50°C; mutants G230K/D113S/D115T and G230T/D113S/D115T exhibited ~5 fold improvement in specific activity.

The engineered TrEGI enzymes also showed higher activity on solid substrates: Avicel and ionic-liquid pretreated Avicel (IL-Avicel) (Additional file 1: Figure S3). All of the engineered enzymes showed ~2-fold higher activity for Avicel hydrolysis (Additional file 1: Figure S3A) and ~5-fold improvement for IL-Avicel hydrolysis (Additional file 1: Figure S3B, G230X/D113S/D115T, X = K, A, S, R) at 50°C relative to the wild-type TrEGI expressed by cell-free synthesis. Many of these TrEGI mutants were active against both substrates at 55°C, conditions under which the wild-type TrEGI expressed by cell-free synthesis was essentially inactive.

### Characterization of *Trichoderma reesei* endoglucanase I variants expressed in *S. cerevisiae*

Considering the impact of proper folding and glycosylation on the stability, activity, and structure of proteins [20-24], we sought to express TrEGI enzymes in a fungal host. Because recombinant expression of proteins in filamentous fungi is typically tedious, time-consuming, and not amenable to high-throughput, we used *S. cerevisiae* [25] as an intermediate fungal expression host to evaluate the stability and activity of the engineered TrEGI triple mutants (enzymes expressed in *S. cerevisiae* are designated Sc_TrEGI). Of all the triple mutants tested (G230X/D113S/D115T, X = K, A, S, R, E, L, T, M), only five of the TrEGI mutants were expressed in active and soluble forms (G230X/D113S/D115T, X = A, E, R, L, T). The enzymes were secreted from yeast in a hyperglycosylated state (MW of 70–200 KDa) as observed previously [26,27], and thus were treated with an endoglycosidase (PNGase F) that cleaves N-glycans, reducing the molecular weight to 54–56 kDa (TrEGI MW is 46 kDa based on aa sequence, with glycans contributing 8–10 kDa), which nearly matches the molecular weight of the natively-expressed TrEGI enzyme (50–55 kDa) [26].

**Figure 4:**
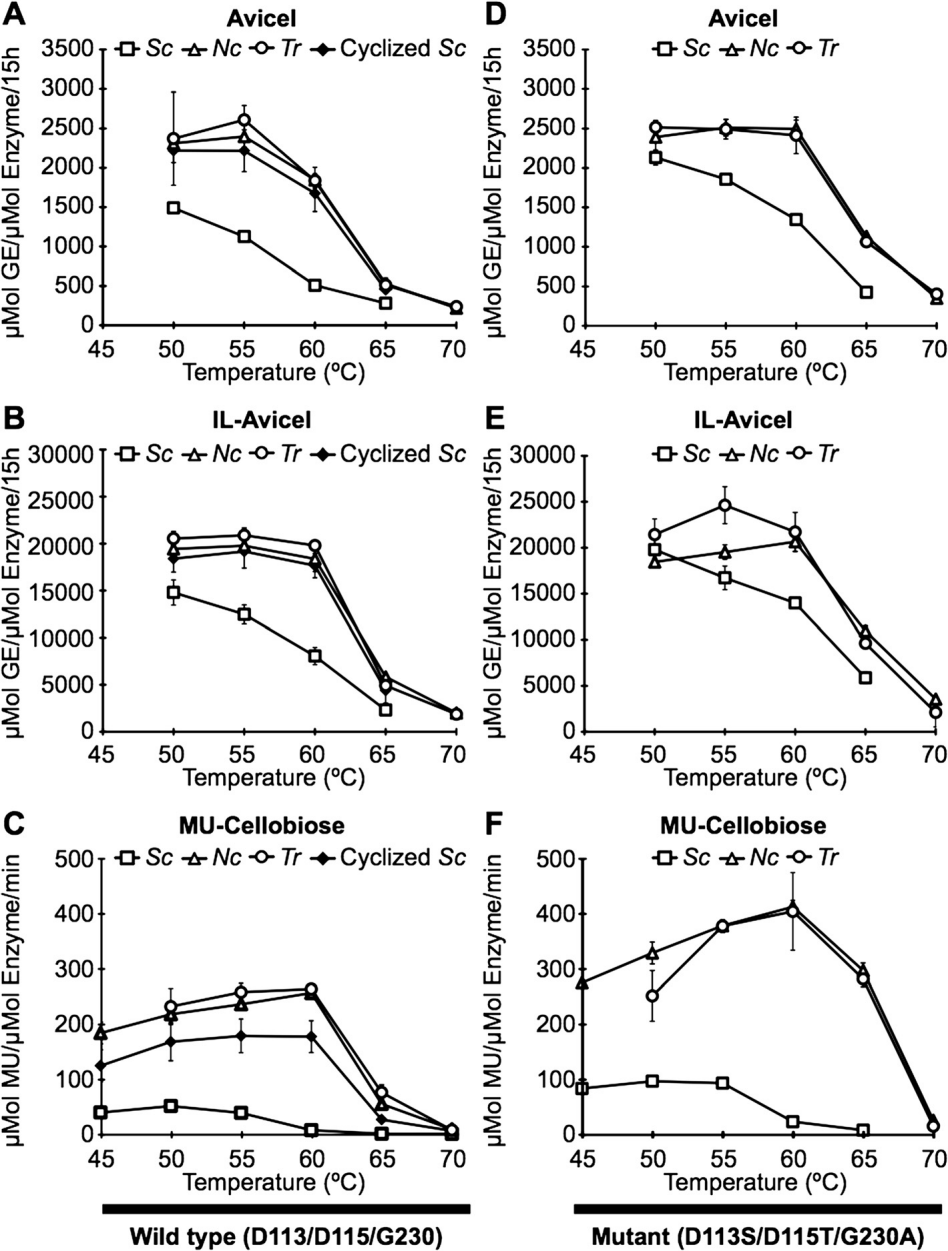
**Temperature activity profile of wild-type** ***T. reesei*** **endoglucanase I (A-C) and G230A/D113S/D115T mutant (D-F) on Avicel (A,D), IL-Avicel (B,E), and MU-Cellobiose (C,F) expressed in** ***S. cerevisiae*** **,** ***N. cra*** **ssa,** ***T. reesei*** **, and** ***S. cerevisiae*** **followed by N-terminal cyclization of the wild-type TrEGI with gutaminyl cyclase.** Error bars represent one standard deviation. Assays were carried out with purified TrEGI proteins (>95% pure based on SDS-PAGE analysis).

Many of these TrEGI mutants exhibited higher hydrolytic activities on soluble (MU-cellobiose) and solid substrates (Avicel and IL-Avicel) compared to Sc_TrEGI (Additional file 1: Figure S4) at 50–65°C. The mutant G230A/D113S/D115T Sc_TrEGI (Figure 4D and 4E) is > 2.5-fold better than Sc_TrEGI (Figure 4A and 4B) at hydrolyzing Avicel at 60°C and IL-Avicel at 65°C. In addition, the activity of G230A/D113S/D115T Sc_TrEGI at 60°C against Avicel and IL-Avicel is similar to that of Sc_TrEGI at 50°C on these substrates. Hence, B-factor guided engineering of TrEGI provided variants with hydrolytic activities on Avicel and IL-Avicel at 60°C similar to the activity of Sc_TrEGI toward these same substrates at 50°C.

All of the engineered triple mutants expressed in *S. cerevisiae* exhibited higher apparent melting temperatures (T_m_ = 58–61°C; measurements were performed as described in Methods using differential scanning calorimetry) than Sc_TrEGI (T_m_ = 57°C). Mutant G230A/D113S/D115T Sc_TrEGI (T_m_ = 61°C, Table 1) showed a ~4°C increase in apparent melting temperature over Sc_TrEGI. No significant difference in half-life at 70°C was observed between Sc_TrEGI and this mutant (Table 1). It is important to note that our initial efforts included expressing the TrEGI enzymes bearing a C-terminal his_6_ tag, which lowered their activity on Avicel (up to 3-fold; compare Additional file 1: Figure S4A and Figure S5), but had little effect on their activity on IL-Avicel (data not shown). This result suggests abrogated binding to Avicel of TrEGI enzymes bearing a C-terminal his_6_ tag.

**Table 1:** **Biochemical characterization of TrEGI expressed in different hosts**

**Expression host**	**Enzyme**	**T**_**m**_ **(°C)**	**t**_**1/2**_ **(hr)**			
			**50°C**	**60°C**	**65°C**	**70°C**
*S. cerevisiae*	WT	57	-	-	-	3 (0.4)
*S. cerevisiae*	G230A/D113S/D115T	61	-	-	-	3.7 (0.5)
*S. cerevisiae*	WT + Glutaminyl Cyclase	65	-	-	-	-
*N. crassa*	WT	65	>5 days	70.6 (3.5)	4.0 (0.2)	3.3 (0.1)
*N. crassa*	G230A/D113S/D115T	68	>5 days	176 (16)	3.3 (0.3)	3.5 (0.4)
*T. reesei*	WT	65	>5 days	74 (2.5)	5.8 (0.6)	6.2 (0.4)
*T. reesei*	G230A/D113S/D115T	68	>5 days	161 (5)	6.2 (0.7)	6.4 (0.3)

T_m_ is the apparent melting temperature for the enzyme, and t_1/2_ is half-life in hours. - = Not determined. Errors are reported in parentheses and represent one standard deviation for measurements. Apparent melting temperature data for Sc_TrEGI (with and without glutaminyl cyclase treatment) and Tr_TrEGI are shown in (Additional file 1: Figure S9). Apparent melting temperature data are not shown for other TrEGI enzymes. Half-life measurements for *T. reesei-* and *N. crassa*-expressed enzymes are shown in (Additional file 1: Figure S6 and Figure S7), respectively. Half-life measurements for *S. cerevisiae*-expressed TrEGI enzymes are not shown.

### Characterization of *Trichoderma reesei* endoglucanase I variants expressed in the filamentous fungi *N. crassa* and *T. reesei*

Sc_TrEGI showed a much lower apparent melting temperature (T_m_ = 57°C) and temperature optimum (50°C) compared to the native TrEGI (T_m_ ~65°C, T_opt_ 60°C) [18], which could be due to non-optimal folding and/or aberrant glycosylation of TrEGI when expressed in *S. cerevisiae*. In order to investigate these possibilities, and to compare the properties (activity and stability) of the engineered TrEGI enzymes to the native TrEGI, we pursued TrEGI expression in *T. reesei*. Thus the wild-type TrEGI and the best mutant (G230A/D113S/D115T TrEGI) were expressed in *T. reesei* QM9414. In addition, our interest in evaluating alternative filamentous fungal systems for cellulase expression prompted us to express TrEGI enzymes (wild-type, G230A/D113S/D115T, and G230E/D113S/D115T) in the filamentous fungus *N. crassa*, which is closely related to *T. reesei.* TrEGI enzymes (wild-type or G230A/D113S/D115T) expressed in *T. reesei* and *N. crassa* were found to have identical temperature optima on MU-cellobiose (Figure 4C, 4F), and apparent melting temperatures (Table 1), which in the case of wild-type enzyme (Tr_TrEGI and Nc_TrEGI) is also identical to the values reported previously for the native TrEGI. Furthermore, little difference was observed between their activities on solid substrates Avicel (Figure 4A) and IL-Avicel (Figure 4B), thus validating *N. crassa* as a suitable surrogate of *T. reesei* for TrEGI production.

G230A/D113S/D115T Tr_TrEGI showed similar or higher activity on solid substrates (Figure 4D and 4E) than Tr_TrEGI when assayed at 50–70°C (Figure 4A and 4B); specifically, a ~2-fold increase in activity was observed at 65°C. In addition, G230A/D113S/D115T Tr_TrEGI showed similar hydrolytic activity on solid substrates at 60°C as the Tr_TrEGI at 50°C. As expected, similar improvements were also observed for *N. crassa*-expressed TrEGI variants. Hence, B-factor guided engineering of TrEGI provided variants with hydrolytic activities on Avicel and IL-Avicel at 60°C similar to the activity of Tr_TrEGI toward these same substrates at 50°C.

G230A/D113S/D115T Tr_TrEGI (T_m_ = 68°C), G230A/D113S/D115T Nc_TrEGI (T_m_ = 68°C), and G230E/D113S/D115T Nc_TrEGI (T_m_ = 68°C) showed a 3°C increase in apparent melting temperature (Table 1 and, Additional file 1: Table S1) compared to Tr_TrEGI or Nc_TrEGI (T_m_ = 65°C). In order to assess whether the thermostable mutants also show improved half-lives over the wild-type TrEGI, half-lives of the thermostable mutant G230A/D113S/D115T TrEGI and the wild-type TrEGI (*T. reesei*- or *N. crassa*-expressed) were measured at 50°C, 60°C, 65°C and 70°C (Table 1 and Additional file 1: Figure S6-S7). Although the half-life for TrEGI enzymes at 50°C was too long (>5 days) to distinguish any gains made at this temperature (data not shown), the G230A/D113S/D115T TrEGI variant showed a >2-fold increase in half-life at 60°C compared to the corresponding recombinant (*T. reesei* or *N. crassa*) wild-type TrEGI (Table 1). The improved half-life (t_1/2_ = 161 hr) of the G230A/D113S/D115T Tr_TrEGI variant at 60°C results in improved or comparable performance of this mutant in biomass saccharification carried out at 60°C compared to the performance of Tr_TrEGI at 50°C (Figure 4A, B, D and E). We also expect that the increased stability and half-life of the G230A/D113S/D115T Tr_TrEGI at temperatures such as 60°C will correlate with improved half-life at lower temperatures (≤60°C) compared to Tr_TrEGI. The half-lives of both these enzymes were significantly shorter (~3–6 hr) (Table 1) at higher temperatures (65°C and 70°C). It is surprising that little or no difference was found between the half-lives of G230A/D11S/D115T TrEGI and the wild-type enzyme (*T. reesei*- or *N. crassa*-expressed) at either 65°C or 70°C (Table 1), which may reflect a shift in the inactivation mechanism(s) with temperature mediated by the different enzyme structures and/or glycosylation patterns as observed previously with other β-sandwich or multi-domain proteins [28,29].

### Impact of N-terminal pyroglutamate on activity and stability of *T. reesei* endoglucanase I

TrEGI and G230A/D113S/D115T TrEGI expressed in both *T. reesei* and *N. crassa* were found to have much higher T_m_ values (7–8°C difference) than their *S. cerevisiae*-expressed counterparts (Table 1). In addition, they were up to 3.6-fold more active on Avicel (Figure 4A and D) at 50–65°C and up to 2.5-fold more active on IL-Avicel (Figure 4B and E) at 50–65°C compared to their counterparts expressed in *S. cerevisiae*. Given the similarity of molecular weights of the TrEGI glycoforms expressed in *S. cerevisiae* and *N. crassa* (Additional file 1: Figure S8), the lower stability and activity of *S. cerevisiae* TrEGI could potentially be due to non-optimal folding (due to protein modifications or lack thereof) and/or incorrect disulfide formation upon expression in *S. cerevisiae*. In this connection, we investigated the role of N-terminal pyroglutamate observed in the *T. reesei* EGI crystal structure.

The available crystal structure of *T. reesei* EGI (pdb code 1EGI) [7] reveals a buried N-terminal pyroglutamate in the natively-expressed enzyme resulting from the cyclization of the N-terminal glutamine. We hypothesized that the lack of this N-terminal glutamine cyclization (confirmed by N-terminal sequencing of *S. cerevisiae-*expressed TrEGI enzyme) in *S. cerevisiae-*expressed enzyme would most likely result in non-optimal protein structure and contribute to the observed decreased stability and activity compared to the *T. reesei*-expressed enzyme. Glutaminyl cyclases (E.C. 2.3.2.5) are known to catalyze the irreversible lactamization of a N-terminal glutamine to a hydrophobic pyroglutamate in many proteins and peptides [30]. Using a human glutaminyl cyclase enzyme [31] we cyclized the N-terminal glutamine residue of Sc_TrEGI to a pyroglutamate, as evidenced by a blocked N-terminal sequence, typical of proteins/peptides with N-terminal pyroglutamate [32].

Treating Sc_TrEGI with the human glutaminyl cyclase substantially increased its activity on solid substrates compared to the corresponding enzyme without glutaminyl-cyclase treatment (Figure 4A-B), and shifted its temperature optimum from 50°C to 60°C on soluble substrates (Figure 4C). The activity of the glutaminyl-cyclase treated Sc_TrEGI on Avicel and IL-Avicel is similar if not identical to its counterpart expressed in *T. reesei* or *N. crassa* (Figure 4A-B).

The increased stability of Sc_TrEGI from N-terminal glutamine cyclization was evaluated by measurements of apparent melting temperatures (Additional file 1: Figure S9). Glutaminyl-cyclase treated Sc_TrEGI (T_m_ = 65°C) showed an ~8°C increase in apparent melting temperature compared to the corresponding uncyclized version (Table 1). The apparent melting temperature of Sc_TrEGI upon cyclization (Table 1) was identical to that of Tr_EGI (T_m_ = 65°C). The large increase in T_m_ upon N-terminal glutamine cyclization indicates that the lower stability of the *S. cerevisiae* enzymes is due to the uncyclized N-terminal glutamine. In addition, it is worth noting that the differences in glycosylation between Sc_TrEGI and Nc_TrEGI (Additional file 1: Figure S8) are irrelevant to the activity and stability differences between these enzymes in comparison to the cyclization of the N-terminal glutamine. Recently our group also demonstrated a similar increase in stability and activity upon cyclization of N-terminal glutamine to pyroglutamate in *S. cerevisiae*-expressed *Talaromyces emersonii* Cel7A [33]. The ubiquitous presence of the N-terminal pyroglutamate in GH7 enzymes (Cel7A and Cel7B), along with its role in imparting high stability/activity for these enzymes, highlights the importance of N-terminal glutamine cyclization for achieving high-efficiency cellulases.

### Impact of mutagenesis and expression host on activity and stability of *T. reesei* endoglucanase I

Combinations of TrEGI mutations at amino acids G230, and D113/D115 identified using the B-factor method resulted in improved TrEGI enzymes irrespective of the expression host for the enzyme. In particular, TrEGI mutant G230A/D113S/D115T showed improved activity on solid substrates as well as improved thermostability for each expression host when compared to wild-type TrEGI enzyme expressed in the same host. These mutations may rigidify the mobile portions of the TrEGI enzyme, and thus enhance its stability. In particular, it is likely that the mutation at G230, which is located on a solvent exposed loop, imparts improved stability by reducing the entropy (rigidifying) of the (partially) unfolded TrEGI, a well-known mechanism for improving protein stability by replacing conformationally flexible residues such as glycine (located in loops or secondary structural elements) with more rigid residues such as alanine [34,35]. Removal of unfavorable electrostatic interactions involving negative charges on D113/D115 in the wild-type enzyme could also lead to increased stability [36]. A small difference in mass was observed between Nc_TrEGI and G230A/D113S/D115T Nc_TrEGI (mass difference = 0–2 Da, Additional file 1: Figure S8 C/D) and between Sc_TrEGI and G230A/D113S/D115T Sc_TrEGI (mass difference = 2–59 Da, Additional file 1: Figure S8 A/B), after accounting for the mass difference resulting from the G230A/D113S/D115T mutation. This shows that there is little or no difference in the extent of glycosylation (total glycan content = 8–10 kDa) between the wild-type TrEGI and the mutant G230A/D113S/D115T TrEGI when they are expressed in the same fungal host. Thus, the improved stability of G230A/D113S/D115T TrEGI compared to wild-type TrEGI when expressed in either *N. crassa* or *S. cerevisiae* is due to the G230A/D113S/D115T mutation and not due to changes in glycosylation.

The specific activity of TrEGI enzymes (wild-type and G230A/D113S/D115T) on solid substrates was dramatically altered upon expression in different hosts (cell-free, *S. cerevisiae, N. crassa,* and *T. reesei*) with *T. reesei* or *N. crassa* producing the most active enzymes on Avicel and IL-Avicel (Figure 4). Glycosylation of TrEGI enzymes renders them more thermostable compared to their cell-free expressed counterparts, which is consistent with previous reports showing the importance of glycosylation in protein thermostability [20-22,24]. The cyclization of N-terminal glutamine to a pyroglutamate in *S. cerevisiae* expressed TrEGI resulted in a TrEGI enzyme with near identical properties to the natively expressed enzyme.

## Conclusions

Using B-factor guided mutagenesis, *T.reesei* endoglucanase I variants were developed that were up to 2-fold more active on solid cellulosic substrates at 65°C than the recombinant wild-type enzyme expressed in the same hosts, and exhibited the same activity at 60°C as the recombinant wild-type enzyme at 50°C. The most stable TrEGI mutant G230A/D113S/D115T showed a ~3°C increase in T_m,_ as well as more than 2-fold improved half-life at 60°C over the native TrEGI. Amino acid changes at G230 coupled with the D113S/D115T double mutation in TrEGI gave rise to variants with improved thermostability and activity at 50–70°C, irrespective of their expression host. However, the stability and activity of the TrEGI enzymes varied significantly when expressed using *E. coli* cell extract, *S. cerevisiae, N. crassa,* or *T. reesei.* TrEGI variants expressed in *N. crassa* or *T. reesei* yielded the most active and stable TrEGI enzymes and *S. cerevisiae* yielded inferior TrEGI enzymes. Cyclization of N-terminal glutamine to a pyroglutamate in *S. cerevisiae*-expressed TrEGI improved its properties to closely match those of the native enzyme, highlighting the importance of this ubiquitous modification in Cel7B enzymes. In this study we show that a non-glycosylating host can be used to screen and discover thermostability imparting mutations in fungal enzymes that result in improved enzymes irrespective of the protein production host. The choice of host for protein engineering of cellulases may not be as critical as the protein production host, which plays a major role in determining the properties of the expressed enzymes.

## Methods

All experiments were carried out using either purified TrEGI enzymes (when expressed in *S. cerevisiae*, *N. crassa,* or *T. reesei*) or TrEGI enzymes expressed via cell-free protein synthesis without any purification. Purity of purified enzymes (>95%) was determined using SDS-PAGE analysis with Coomassie blue dye staining.

### Construction of plasmid bearing *T. reesei* endoglucanase I

Gene for the *T. reesei* endoglucanse I (*cel7b*, UniProt No. P07981) synthesized with *E. coli* codon bias was purchased from GenScript (Genscript Corporation, Piscatway, NJ, USA). The structural gene of cel7b was PCR-amplified from plasmid pUC57-P07981 by using the forward primer (5’-AAAAAACATATG CAACAACCGGGCACCTCC-3′) and reverse primer (5′-AAAAAAGTCGACTTACAGACATTGCGAGTAGTA-3′). PCR product was then cloned into pIVEX2.4d vector (Roche Applied Science) after a double digestion with NdeI and SalI to generate plasmid, pIVEX2.4d-TrEG1.

### Preparation of S30 cell-extract

The cell-extracts were prepared as reported previously [16]. To exhaust the reducing activity of cell-extract, the S30 extract was incubated with 10 mM oxidized glutathione for 2 hrs at 30°C. The residual glutathione molecules were removed by dialyzing the treated extract against 200 volumes of buffer C (buffer A without DTT and 2-ME) for 3 hrs at 4°C. The resulting extract was divided into small aliquots and stored at −80°C before using it for cell-free protein synthesis.

### Site directed mutagenesis of *T. reesei* endoglucanase I

Site directed mutagenesis at amino acids 238, 291, and 323 of *T. reesei* EGI (pIVEX2.4d-TrEG1 as template plasmid) was performed using the site directed mutagenesis kit (Stratagene) by following the manufacturers protocol with primers shown in Additional file 1: Table S2. Mutagenesis at sites with amino acids 230, 113 and 115, 284–287, and 301–302 of *T. reesei* EGI (pIVEX2.4d-TrEG1 as template plasmid) was performed using Overlap Extension PCR. All PCR reactions were carried out using Pfu DNA polymerase (Stratagene, La Jolla, CA). The plasmids were transformed into electrocompetent XL1 Blue cells and plated. Colonies were picked and transferred into 96-well plates using a colony picker (Qpix) and grown overnight at 37°C in 10% glycerol containing LB medium. For site A, ~3000 colonies were picked, corresponding to ~2% coverage; for all other sites, colonies corresponding to >95% coverage of sequence space were picked and grown overnight in 96-well plates (~3000 colonies for sites B and C, and ~500 colonies for sites D-G).

### Cell-free protein synthesis

Overnight culture bearing the mutant *T. reesei* EGI gene was used as a template for the PCR reaction. TrEGI DNA amplified by PCR using T7 fwd and T7 rev primers served as template for protein synthesis. The standard reaction mixture for cell-free protein synthesis consisted of the following components in a total volume of 15 μL: 57 mM of Hepes-KOH (pH 7.5); 1.2 mM of ATP; 0.85 mM each of CTP, GTP, and UTP; 0.17 mg/mL of *E.coli* total tRNA mixture (from stain MRE600); 90 mM of potassium glutamate; 80 mM of ammonium acetate; 12 mM of magnesium acetate; 34 μg/mL of L-5-formyl-5, 6, 7, 8-tetrahydrofolic acid (folinic acid); 2.0 mM each of all 20 amino acids; 2% PEG (8000); 67 mM of creatine phosphate (CP); 3.2 μg/mL of creatine kinase (CK); 2.5 mM oxidized glutathione; 2.5 mM reduced glutathione; 16.7 μg/mL of PCR product; 27%(v/v) of S30-GroEL/ES cell-extract. The cell-free reaction was conducted at 30°C for 2 hours. For determining the amount of the protein synthesized using the cell-free synthesis, 10 μM of L-[U-^14^C] Leucine (11.3 GBq/mmol, Amersham Biosciences) was also added to the cell-free reaction. To analyze the solubility of cell-free expressed protein, the cell-free mixture was centrifuged at 15,000 rpm for 10 min at 4°C. The soluble amount of synthesized protein was determined by analyzing the supernatant of the centrifugation. The amount of each cell-free synthesized cellulase was estimated from the TCA-insoluble radioactivity using a liquid scintillation counter (Tri-Carb 2810TR Liquid Scintillation Analyzer, Perkin-Elmer Inc.).

### Screening *T. reesei* endoglucanase I mutants for improved thermostability/activity

Two μL of 1 M sodium acetate buffer pH 4.85 was added to 15 μL of cell-free expressed TrEGI mutant to lower the pH to 5. TrEGI mutant was then heated to 50°C for 30 mins after which 1 μL was spotted on CMC-agar plate (1%CMC, 1.5% agar, 50 mM pH 4.85 Acetate buffer) and incubated overnight (12–16 hr) at 50°C. The CMC-agar plates were stained with 15 mL of 1% congo red for 5 mins. The plates were then analyzed for presence of clearing zones, which represented active enzymes (hits). All experiments were carried out using TrEGI enzymes expressed using cell-free protein synthesis without any purification.

### Measuring the thermostability of *Trichoderma reesei* endoglucanase I

To measure the thermostability of TrEG1, 10 μL of the cell-free synthesized TrEG1 (20 pmol) was mixed with 10 μL of 200 mM of sodium acetate buffer (pH 4.85). After incubating the enzyme mixture at 50°C with different incubation time points (0, 15, 30, 45, 60 min), 80 μL of 1.25% (w/v) CMC containing 100 mM sodium acetate buffer (pH 4.85) was added into the heat-pretreated enzyme mixture to start hydrolysis reaction for 1 hr at 50°C. The reducing sugar concentration was measured by the DNS assay as described previously [16]. All experiments were carried out in triplicate using TrEGI enzymes expressed via cell-free protein synthesis without any purification.

### Endoglucanase activity measurement on solid cellulosic substrates

All cellulolytic assays for solid substrates were carried out in quadruplicate in 96-well plates in a final volume of 70 μL containing 1% (w/v) substrate, 100 mM sodium acetate buffer pH 4.85, 0.2 μM of the cellulase at 50–70°C. The 96-well plates were sealed with aluminum foil and incubated without shaking in a thermocycler with a heated top to minimize evaporation. Cellulase activities were measured for Avicel, and ionic-liquid pretreated Avicel (IL-Avicel). The mixtures were incubated at 50–70°C for 15 h after which they were cooled to 4°C prior to measuring the amount of soluble reducing sugar released using the glucose oxidase-peroxidase assay as described previously [16]. IL-avicel was prepared as described previously [16]. All experiments were carried out using either purified TrEGI enzymes (when expressed in *S. cerevisiae*, *N. crassa,* or *T. reesei*) or TrEGI enzymes expressed via cell-free protein synthesis without any purification. Enzyme purity (>95%) was determined using SDS-PAGE analysis with Coomassie blue dye staining.

### Expression of *T. reesei* endoglucanase I in *S. cerevisiae*

For production of *T. reesei EGI* and engineered mutants in *S. cerevisiae*, the corresponding genes (*E. coli* codon optimized as described above) were amplified from pIVEX 2.4d vector and were cloned with and without a his6 tag on the C-terminus in pCu424 vector [37]. An engineered α-factor AppS4 [38] prepro leader sequence was appended onto the N-terminus of the genes to enable secretion of the enzyme. pCu424 containing *T. reesei EG1* gene was transformed into *S. cerevisiae* strain YVH10 [39] with additional *PMR1* knockout using the LiAc method [40]. For expression, a saturated YPD medium preculture was used to inoculate 1 L selective medium (SC-Trp) and grown for three days at 30°C. The culture was spun down at 4000 g for 5 min and resuspended in YPD medium supplemented with 500 μM CuSO_4_ for a three-day induction at 25°C. Culture supernatant was concentrated to ~100 mL (10 kDa cut-off) prior to purification. TrEGI bearing the his6 tag was purified from culture supernatant using Ni-affinity chromatography. TrEGI without the his6 tag was purified from culture supernatant as described below for *N. crassa* TrEGI.

### Deglycosylation of *T. reesei* endoglucanase I expressed in *S. cerevisiae*

Three to five mg of purified *T. reesei* EG1 expressed in *S. cerevisiae* was incubated in pH 7.0, 25 mM phosphate buffer along with 200 units of PNGase F (NEB) at 30°C for 18 h following which an additional 100 units of PNGase F were added and incubated for another 24 h. PNGase F treated *T. reesei EG1* was purified using gel filtration chromatography.

### Temperature optima of *T. reesei* endoglucanase I expressed in *S. cerevisiae*, *N. crassa*, and *T. reesei*

To measure the temperature optima, TrEG1 and the mutants expressed in *S. cerevisiae* (glycosylated versions) and *T. reesei* were assayed for 10 min at different temperatures (40°C–65°C) using 1 mM MU-cellobiose in 100 mM sodium acetate buffer (pH 4.85) as the substrate in a 100 μL reaction volume in a PCR thermocycler. The reaction was quenched by adding 5 μL of the reaction mixture to 145 μL 110 mM CAPS buffer, pH 10.8. Activity was determined by measuring the fluorescence of methyl umbelliferone released. The TrEGI enzyme concentration ranged from 0.1–0.5 μM for initial-rate measurements. All experiments were carried out in quadruplicate. All experiments were carried out using purified enzymes.

### Half-life measurements of *T. reesei* endoglucanase I expressed in *S. cerevisiae*, *N.crassa*, and *T. reesei*

TrEGI enzymes (1 μM) were incubated in 100 mM sodium acetate buffer pH 4.85 at 50°C, 60°C, 65°C and 70°C in a PCR thermocycler. Aliquots were withdrawn at different times and incubated at 4°C for 20 min. Endoglucanase activity was measured at 50°C using MU-cellobiose as described above. The enzyme half-life was calculated by fitting the activity data to exponential or linear functions. All experiments were carried out in duplicate. All experiments were carried out using purified enzymes.

### Cloning, expression and purification of *T. reesei* endoglucanase I in *N. crassa*

*T. reesei* EGI and engineered mutants were expressed in *N. crassa* as described previously [33]. The wild-type TrEGI gene was amplified from *T. reesei* gDNA. The engineered TrEGI mutants were constructed using site-directed mutagenesis kit from Stratagene as described above. TrEGI was purified from culture supernatant in a series of steps. TrEGI was precipitated from the culture supernatant with 65% ammonium sulfate. The supernatant was spun down and the precipitated proteins were resuspended 20 mM Tris–HCl pH 7 buffer and desalted using HiPrep 26/10 desalting column with 20 mM Tris–HCl pH 7 as the running buffer. The protein was then concentrated and loaded on Mono Q column and TrEGI was eluted using 20 mM Tris–HCl pH 7 buffer between 25–75 mM NaCl. TrEGI containing fractions were pooled and further polished using size exclusion chromatography to give pure protein.

### Cloning, expression and purification of *Trichodermareesei* endoglucanase I in *T. reesei*

For production of *T. reesei* EGI and engineered mutants a EGI expression vector was constructed by introducing the EGI gene followed by the *N. crassa* cbhI terminator behind the cdna1 promoter in pPcdna1 expression plasmid (generous gift from Bernhard Seiboth) [41]. Transformation of *T. reesei* by electroporation was adapted as described previously [42]. Spores of *T. reesei* QM9414 were harvested from a 90 mm potato dextrose agar (PDA) plate and suspended in 1.1 M ice cold sorbitol. Spores were washed twice with 1.1 M ice cold sorbitol, pelleted at 800xg for 4 min, and then re-suspended in 100 uL of 1.1 M ice cold sorbitol. Two micrograms of purified PCR product of the expression cassette using the primers 5′- GGATCCGAGAGCTACCTTACATC -3′ and 5′- CGAACTACCTCGCGAAACTCG -3′ was added to the spore suspension and incubated on ice for 30 min. Electroporation was conducted using a Gene Pulser Xcell (Bio-Rad Laboratories, Inc., Hercules, CA) in 1 mm gap cuvette at 1.6 kV, 600 Ω and 25 μF. Immediately following electroporation, 900 μL of 1.1 M ice cold sorbitol was added and gently mixed. The spore suspension was then added to 9 mL of YPD (1% (w/v) yeast extract, 2% (w/v) peptone, 2% (w/v) dextrose) and incubated for 12 hr at room temperature. Spores were pelleted at 800xg for 4 min, re-suspended in 1 mL of 1.1 M sorbitol, and plated using the overlay method onto Mandels-Andreotti medium containing 200 μg/mL hygromycin B. Both top and bottom agars contained hygromycin B. Germinated spores were picked onto PDA slants containing hygromycin B. Positive transformants were selected by presence of EGI activity (AZO-CM-Cellulose Assay, Megazyme, Ireland) in the supernatant after growth in Mandels-Andreotti medium supplemented with 3% glucose, in which QM9414 does not secrete native cellulases [41,43] for 3–4 days. Culture broths were filtered through glass microfiber filters (934-AH, Whatman) followed by 0.22 μm PES filters (Corning). TrEGI was purified from culture supernatant as described above for *N. crassa* TrEGI.

### Mass spectrometry of intact TrEGI protein

Mass Spectra were obtained via an Agilent 6510 Quadrupole Time-of-Flight (Q-TOF) mass spectrometer immediately following external mass calibration. TrEG1 protein samples were diluted in water to a final concentration of 0.75 μM and 1 μL was injected using an autosampler. Samples were analyzed with an Agilent HPLC-chip cube using a custom HPLC-chip (SPQ381) containing Zorbax 300A SB-C3 (5 μm) separation (43 mm × 75 μm) and enrichment (4 mm, 40 nL) columns. Proteins were eluted under a gradient of 3% to 95% Solvent B over 9 min. Solvent A was water with 0.1% formic acid; Solvent B was acetonitrile with 0.1% formic acid. Solvent reagents were of LC/MS grade. The Q-TOF mass spectrometer was run under Extended Dynamic Range (2GHz). The HPLC- Chip voltage was 1.85 kV, gas temperature was 300°C, and drying gas flow was 4 L/min. One spectra was acquired per second with 1000 ms/spectrum using a mass range of 300–3,000 m/z. Data analysis was performed using Agilent Mass Hunter Qualitative Analysis software. Mass Spectra extracted from chromatogram peaks were deconvoluted using the maximum entropy setting with a mass range of 20–80 kDa, mass step of 1 Da, S/N threshold of 30, minimum consecutive charge states of 5, minimum protein fit score of 8, and an average mass of 90% peak height.

### Glutaminyl Cyclase Treatment of *S. cerevisiae* Expressed TrEGI

Lyophilized human glutaminyl cyclase was purchased from Sino Biological (Daxing, China; 13752-H07B) and reconstituted to 0.2 mg/mL. Cyclization was carried out using 0.02 mg/mL cyclase and 50–100 μM TrEGI in 50 mM sodium phosphate pH 7.4 for 18–48 h at 30°C. N-terminal edman sequencing was performed at University of Califonia-Davis, proteomics core facility. All experiments were carried out using purified TrEGI.

### Differential scanning calorimetry

The apparent melting temperature of TrEGI (0.5–1.0 mg/mL) in 50 mM sodium acetate, pH 4.85, was measured using a TA Instruments NanoDSC. After equilibration, a temperature schedule from 25°C to 80°C was employed using a ramp rate of 1°C/min. Raw data are presented for some TrEGI variants (Additional file 1: Figure S9) and the apparent melting temperature corresponds to the apex of each peak. For all TrEGI enzymes mentioned herein, melting was irreversible. All experiments were carried out using purified enzymes.

## Additional file

Additional file 1:
**Includes Figures S1-S9 and Tables S1-S2.**

## Abbreviations

TrEGI: *Trichoderma reesei* endoglucanase I
EGI: Endoglucanase I
Sc_TrEGI: Wild-type *Trichoderma reesei* endoglucanase I expressed recombinantly in *Saccharomyces cerevisiae*
Nc_TrEGI: Wild-type *Trichoderma reesei* endoglucanase I expressed recombinantly in *Neurospora crassa*
Tr_TrEGI: Wild-type *Trichoderma reesei* endoglucanase I expressed recombinantly in *Trichoderma reesei* QM9414
CD: Catalytic Domain
CBM: Carbohydrate Binding Module
CMC: Carboxy Methyl Cellulose
DSC: Differential Scanning Calorimetry
MU-cellobiose: 4-Methyl Umbelliferone-β-D-Cellobioside
PASC: Phosphoric Acid Swollen Celulose
pNPC: *p*-Nitrophenyl-β-D-cellobioside
IL-Avicel: Ionic-liquid pretreated Avicel
$\varDelta {T}_{50}^{60}$: Change in temperature at which the enzyme loses half its activity after an hour of incubation
