# Supplementary material for: Mutagenesis of Trichoderma reesei endoglucanase I: impact of expression host on activity and stability at elevated temperatures
Source: BMC Biotechnol. 2015 Feb 21;15(1):11. doi: 10.1186/s12896-015-0118-z (PMC4347658; doi:10.1186/s12896-015-0118-z)
Supplement: Additional file 1: — Includes Figures S1-S9 and Tables S1-S2. [file 12896_2015_118_MOESM1_ESM.pdf]

Additional File 1

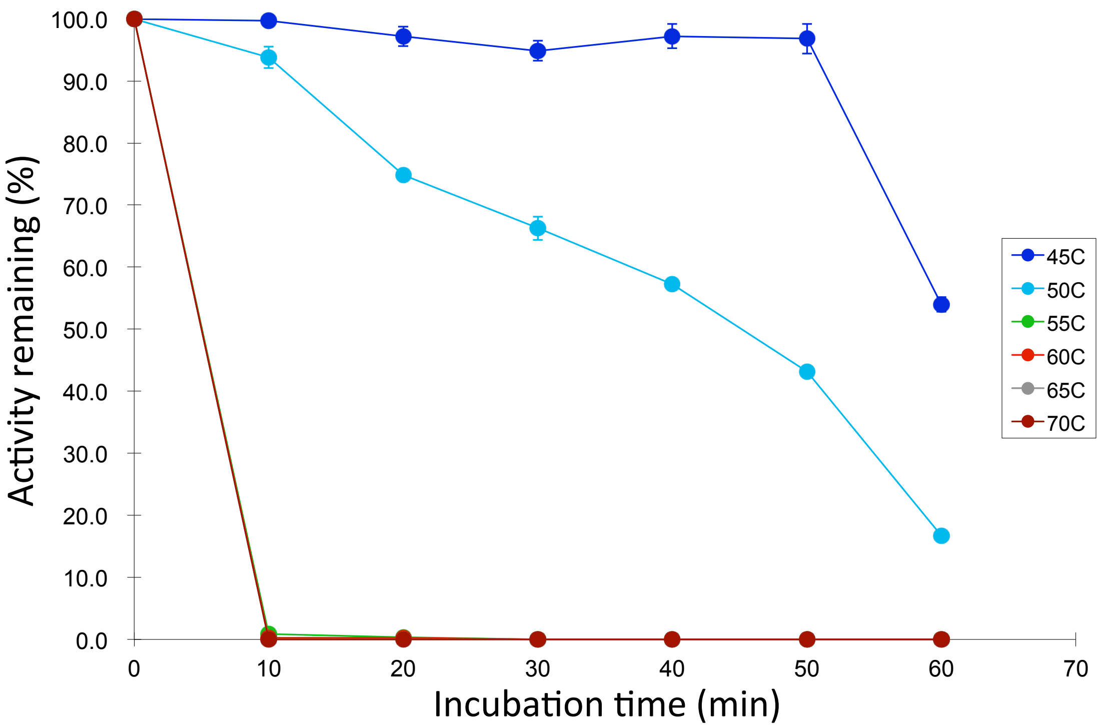

Figure S1 Thermostability of cell-free expressed *Trichoderma reesei* endoglucanase I. The cell-free expressed *T. reesei* EGI was incubated at various temperatures ranging from 45-70°C (legend) prior to measuring activity on CMC. Since the enzyme lost all activity upon incubation at or above 55°C for 10 min, the data points for incubations at 55°C, 60°C, 65°C and 70°C (for 0-60 min) overlap each other in Figure S1. Error bars represent one standard deviation for measurements.

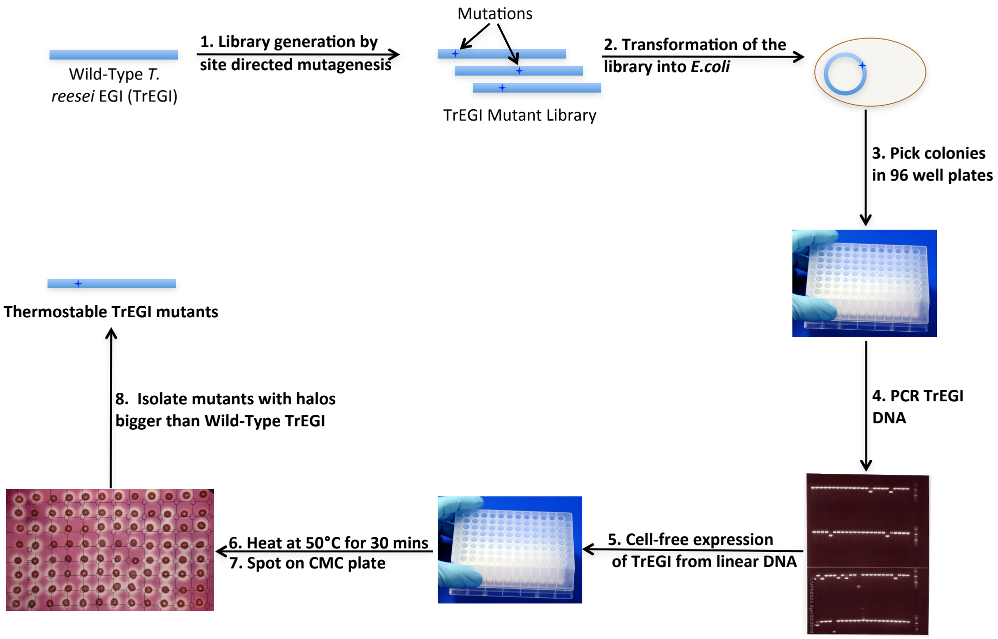

Figure S2 Mutagenesis, screening, and selection of *Trichoderma reesei* endoglucanase I library.

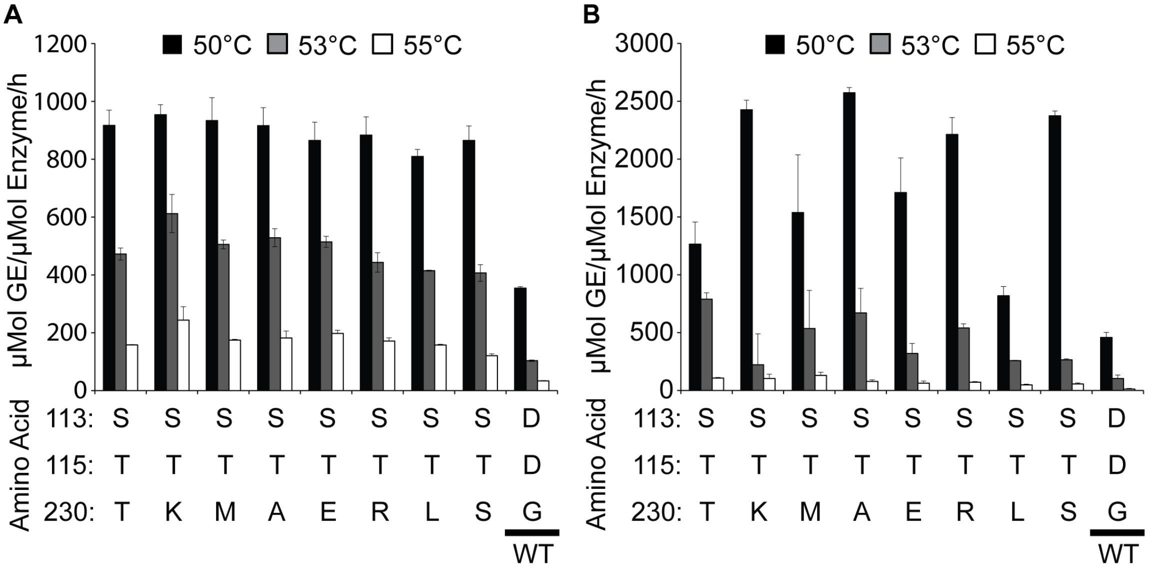

Figure S3 Activity of cell-free expressed TrEGI enzymes on Avicel (A), and IL-Avicel (B). Error bars represent one standard deviation for measurements.

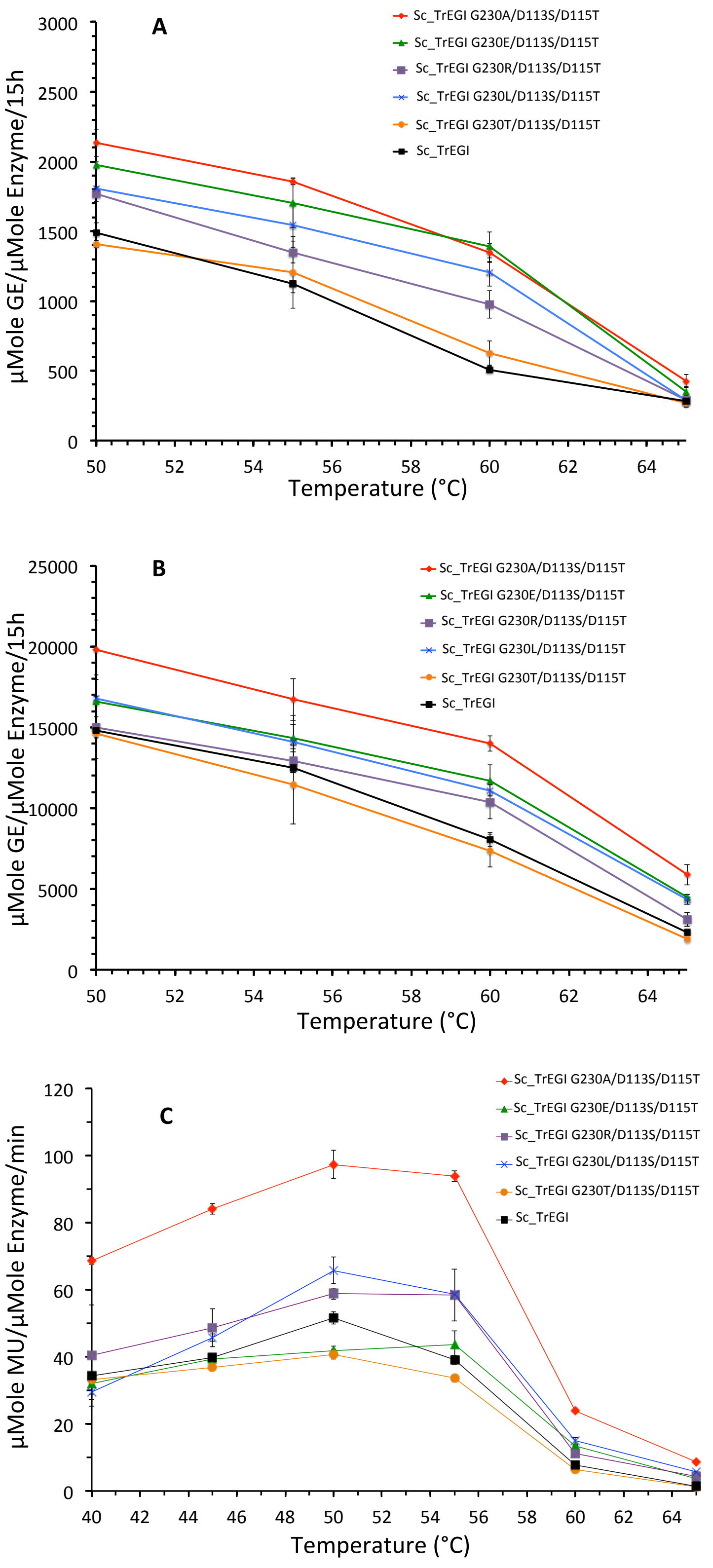

Figure S4 Temperature-activity profiles of *T. reesei* EGI mutants expressed in *S. cerevisiae* on Avicel (A), IL-Avicel (B), & MU-cellobiose (C). Error bars represent one standard deviation for measurements.

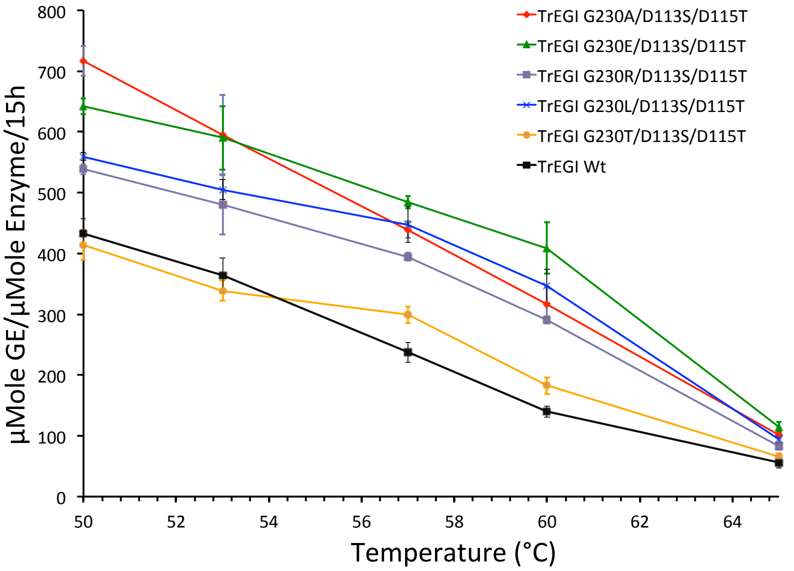

Figure S5 Temperature-activity profiles on Avicel of *T. reesei* EGI mutants expressed in *S. cerevisiae* bearing a C-terminal His6 tag. Error bars represent one standard deviation for measurements.

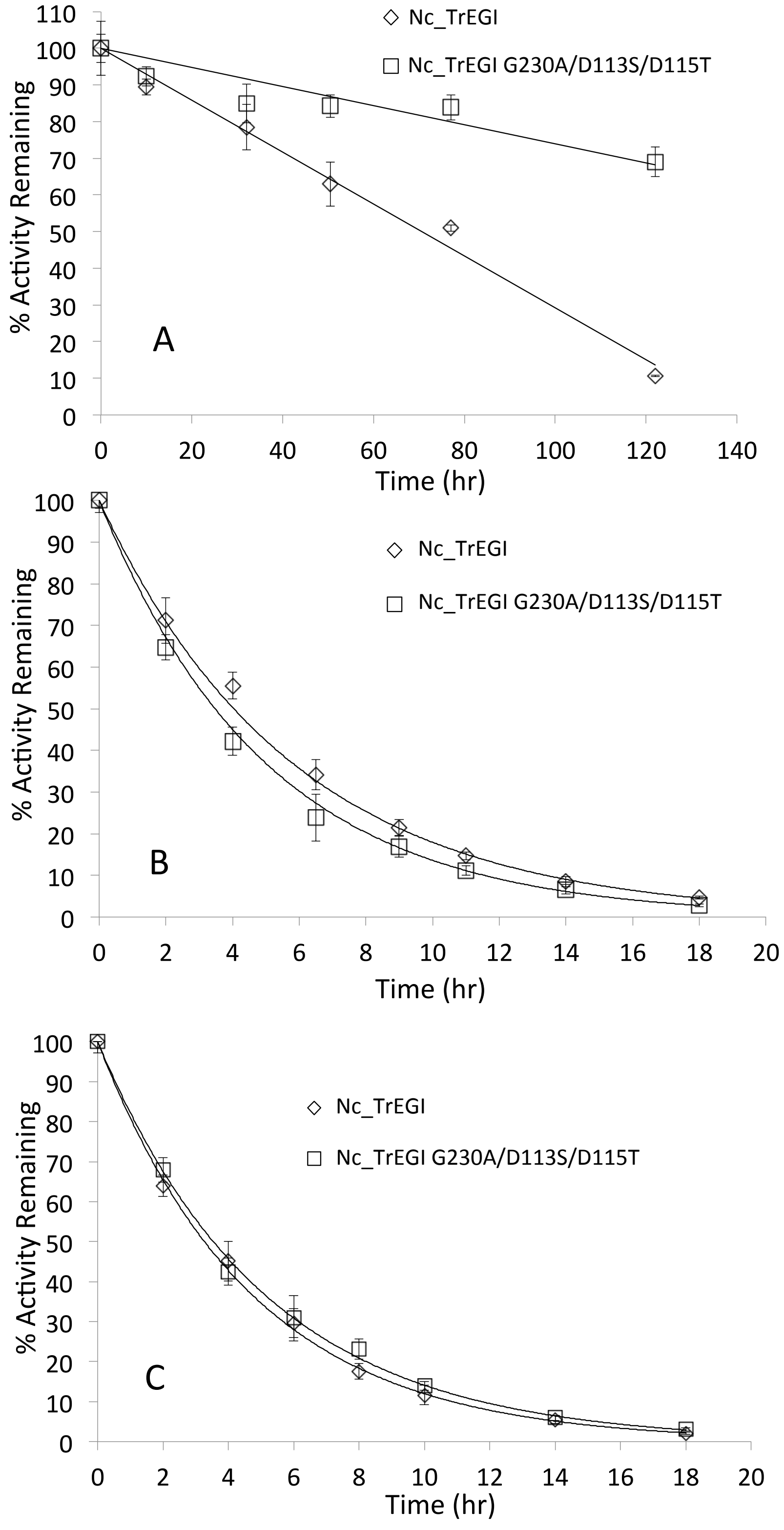

Figure S6 Inactivation of Nc_TrEGI G230A/D113S/D115T and Nc_TrEGI measured at different temperatures: 60°C (A), 65°C (B), and 70°C (C). Error bars represent one standard deviation for measurements.

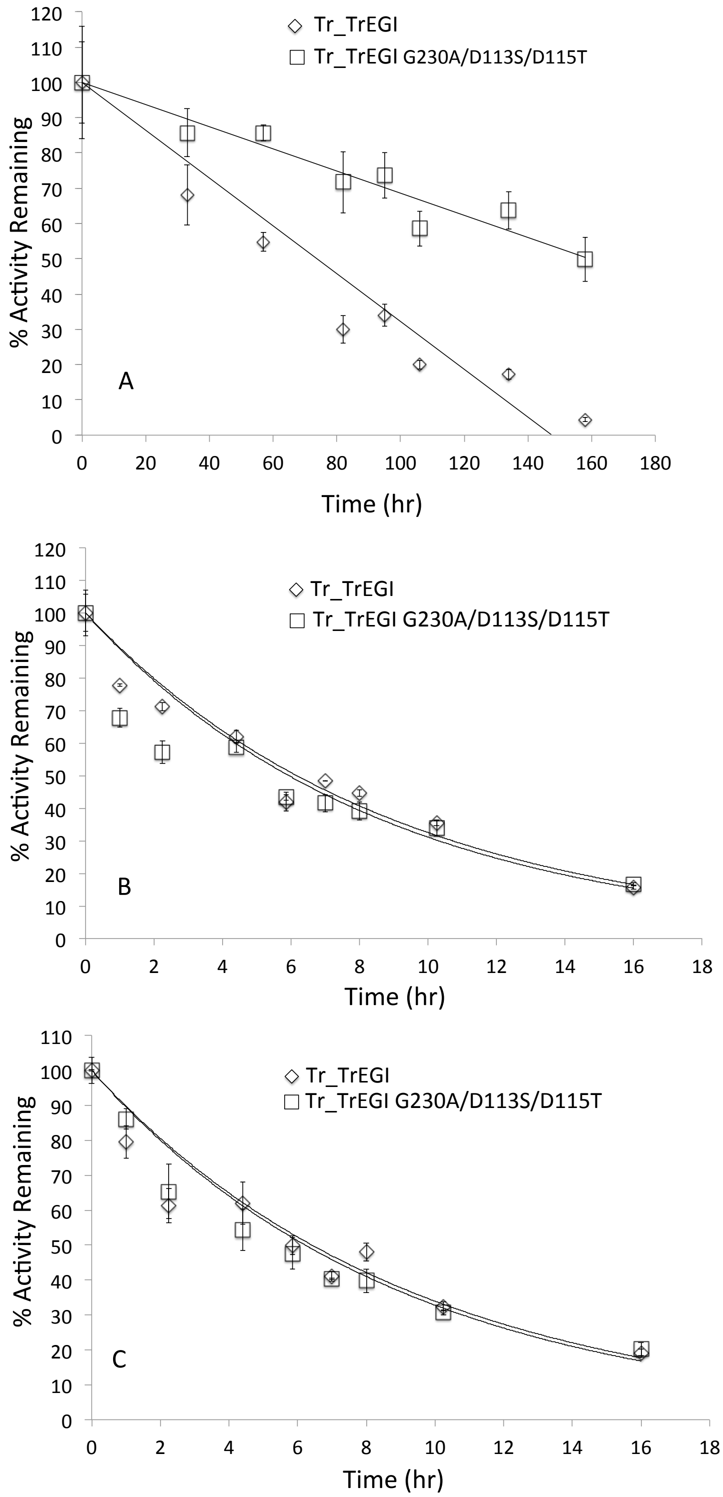

Figure S7 Inactivation of Tr_TrEGI G230A/D113S/D115T and Tr_TrEGI measured at different temperatures: 60°C (A), 65°C (B), and 70°C (C). Error bars represent one standard deviation for measurements.

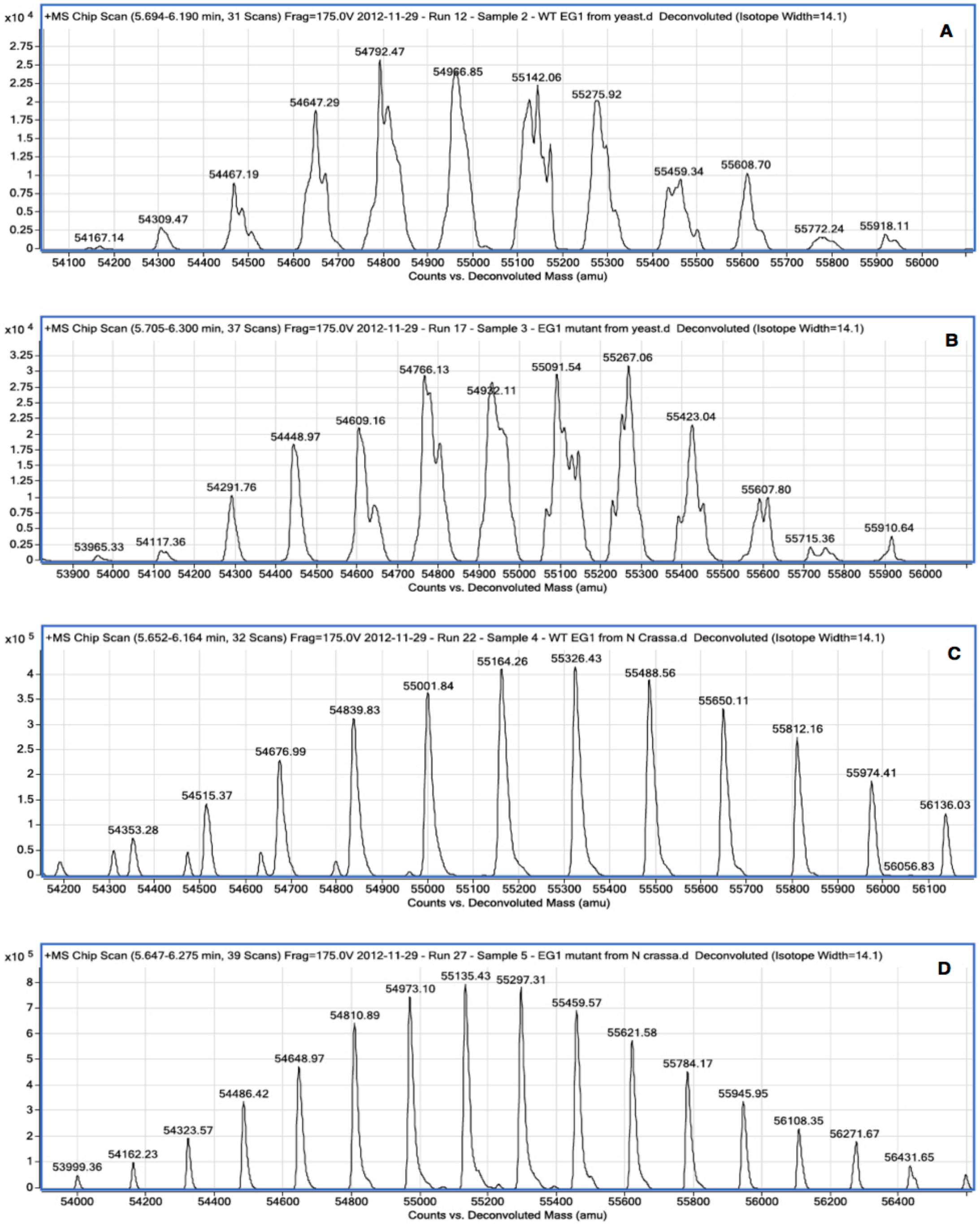

Figure S8 Mass profiles for TrEGI wild-type (MW = 46.002 kDa) and mutant G230A/D113S/D115T (MW = 45.974 kDa) expressed in *S. cerevisiae* and *N. crassa*. MW was calculated from the amino acid sequence of the protein. **A**: MS of Sc_TrEGI; **B**: MS of Sc_TrEGI G230A/D113S/D115T; **C**: MS of Nc_TrEGI; **D**: MS of Nc_TrEGI G230A/D113S/D115T. *S. cerevisiae*-expressed TrEGI enzymes were treated

with PNGase F prior to carrying out LC-MS. Each TrEGI protein exists as a series of glycoforms, with an average mass difference between each individual glycoform of ~162 Da, which corresponds to the MW of a single monosaccharide on the glycan. Comparing the mass of each individual glycoform for Nc_TrEGI (Figure S8, C) with that of the corresponding G230A/D113S/D115T Nc_TrEGI glycoform (Figure S8, D) reveals no appreciable mass difference (mass difference = 0-2 Da) after accounting for the 28 Da mass reduction due to the G230A/D113S/D115T mutation. For *S. cerevisiae*-expressed TrEGI enzymes, microheterogeneity was observed within glycoforms, rendering a precise mass comparison difficult. However, even with the microheterogeneity, observed mass differences between the most abundant glycoforms of Sc_TrEGI (Figure S8, A) and G230A/D113S/D115T Sc_TrEGI (Figure S8, B) range from 2-59 Da (after accounting for the mass change due to the G230A/D113S/D115T mutation), suggesting similar glycan content for these proteins.

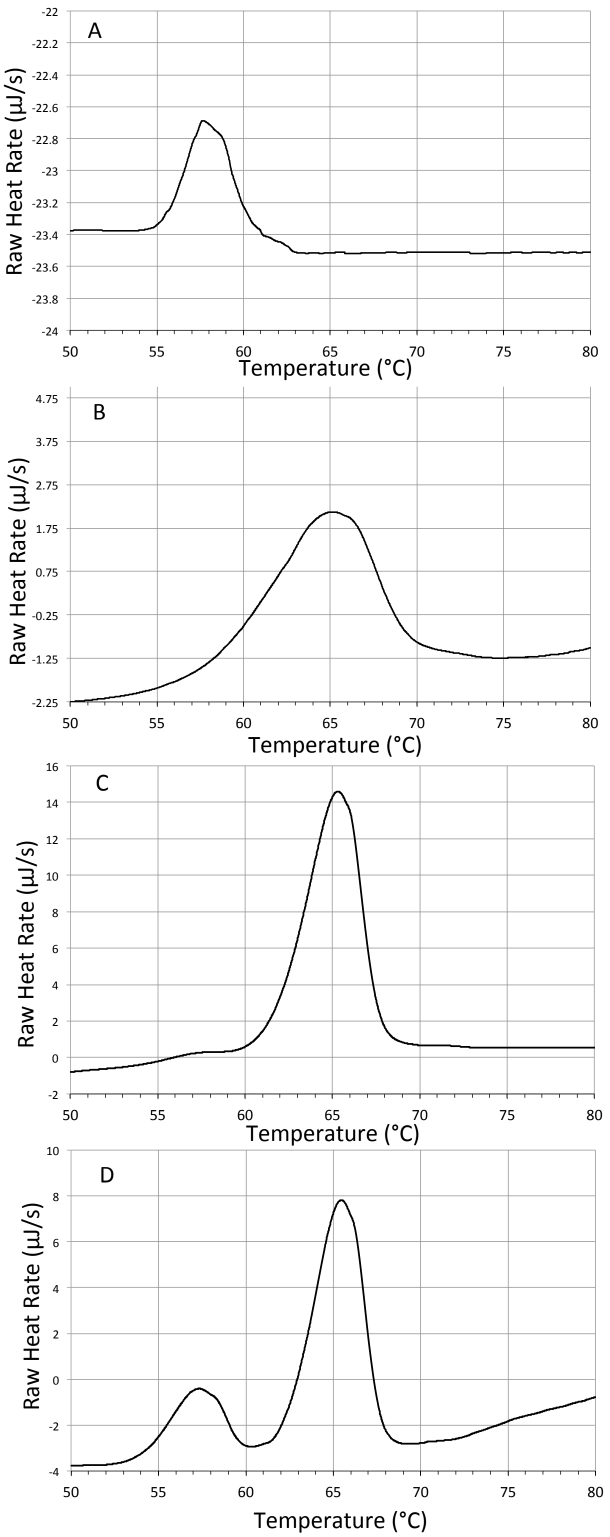

Figure S9 Digital Scanning Calorimetry profiles for wild-type TrEGI expressed in *S. cerevisiae* and *T. reesei.* **A**: DSC of Sc_TrEGI; **B**: DSC of Tr_TrEGI; **C**: DSC of Sc_TrEGI treated with glutaminyl cyclase; **D**: DSC of mixture of Sc_TrEGI and Tr_TrEGI.

Table S1 Biochemical characterization of TrEGI expressed in different hosts. Specific activity was measured at 50°C against all substrates and is reported in µmole glucose equivalents per hour per µmole enzyme. T_m is the meting temperature for the enzyme. – (Not determined). Errors are reported in parentheses. Errors represent one standard deviation for measurements.

Expression Host	Enzyme	CMC	Avicel	IL-Avicel	MU-Cellobiose	T_m (°C)
E. coli cell-extract	TrEGI	3312 (343)	23.6 (0.2)	305 (29)	-	-
E. coli cell-extract	G230A/D113S/D115T TrEGI	12646 (128)	61.1 (4.2)	1716 (30)	-	-
E. coli cell-extract	G230E/D113S/D115T TrEGI	10711 (1605)	57.7 (4.2)	1140 (200)	-	-
S. cerevisiae	TrEGI	-	99.2 (5)	986 (89)	3096 (108)	57
S. cerevisiae	TrEGI treated with glutaminyl cyclase	-	148 (10)	1226 (95)	10084 (2609)	65
S. cerevisiae	G230A/D113S/D115T TrEGI	-	142.2 (6.3)	1319 (122)	5841 (106)	61
S. cerevisiae	G230E/D113S/D115T TrEGI	-	131.8 (1.3)	1105 (110)	1877 (59)	60
N. crassa	TrEGI	-	154.0 (4.3)	1295 (2)	12,972 (37)	65
N. crassa	G230A/D113S/D115T TrEGI	-	159.5 (14.3)	1428 (113)	19,596 (1196)	68
N. crassa	G230E/D113S/D115T TrEGI	-	139.3 (5.5)	1185 (21)	6272 (318)	68
T. reesei	TrEGI	-	158 (40)	1368 (50)	13,913 (1947)	65
T. reesei	G230A/D113S/D115T TrEGI	-	167.8 (2.5)	1428 (91)	15,111 (2747)	68

Table S2 List of primers used for constructing the mutant TrEGI library.

Primer Name	Mutation Site	Sequence
A-FWD	A	GGC GTC GAT ATT CCG TCC GCA NDT NDT NDT NDT GAC ACC ATC TCT AGT TGC CCG TCA GC
A-REV	A	GCT GAC GGG CAA CTA GAG ATG GTG TCA HMA HMA HMA HMT GCG GAC GGA ATA TCG ACG CC
B-FWD	B	TCA GCA TCG GCC TAC GGC NNK NNK GCA ACC ATG GGC AAA GCT C
B-REV	B	GAG CTT TGC CCA TGG TTG CMN NMN NGC CGT AGG CCG ATG CTG A
C-FWD	C	CGC GTC TGT ATC TGC TGN NKT CTN NKG GCG AAT ACG TGA TGC TG
C-REV	C	CAG CAT CAC GTA TTC GCC MNN AGA MNN CAG CAG ATA CAG ACG CG
D-FWD	D	CAA ATC CTA TTA CNN KCC GGG TGA TAC C
D-REV	D	GGT ATC ACC CGG MNN GTA ATA GGA TTT G
E-FWD	E	GTT TCA ACC CGT ATN NKA GTG GTT ACA AAT C
E-REV	E	GAT TTG TAA CCA CTM NNA TAC GGG TTG AAA C
F-FWD	F	CAA TTT GGA ACG ATN NKT CGC AGT ATA TG
F-REV	F	CAT ATA CTG CGA MNN ATC GTT CCA AAT TG
G-FWD	G	GAC ACC ATC TCT NNK TGC CCG TCA G
G-REV	G	CTG ACG GGC AMN NAG AGA TGG TGT C
T7-FWD	Universal FWD	TCG ATC CCG CGA AAT TAA TAC GAC TCA CTA TAG GG
T7-REV	Universal REV	CAA AAA ACC CCT CAA GAC CCG TTT AG
